# Pore-forming activity of new conjugate antibiotics based on amphotericin B

**DOI:** 10.1371/journal.pone.0188573

**Published:** 2017-11-29

**Authors:** Svetlana S. Efimova, Anna N. Tevyashova, Evgenia N. Olsufyeva, Evgeny E. Bykov, Olga S. Ostroumova

**Affiliations:** 1 Group of Ion Channel Modeling, Institute of Cytology of the Russian Academy of Sciences, St. Petersburg, Russia; 2 Laboratory of Chemical Transformation of Antibiotics, Gause Institute of New Antibiotics of the Russian Academy of Medical Sciences, Moscow, Russia; 3 D.I. Mendeleev University of Chemical Technology of Russia, Moscow, Russia; University of Cambridge, UNITED KINGDOM

## Abstract

A series of amides of the antifungal antibiotic amphotericin B (AmB) and its conjugates with benzoxaboroles was tested to determine whether they form pores in lipid bilayers and to compare their channel characteristics. The tested derivatives produced pores of larger amplitude and shorter lifetime than those of the parent antibiotic. The pore conductance was related to changes in the partial charge of the hydrogens of the hydroxyl groups in the lactone ring that determined the anion coordination in the channel. Neutralization of one of the polar group charges in the AmB head during chemical modification produced a pronounced effect by diminishing the dwell time of the polyene channel compared to modification of both groups. In this study, compounds that had a modification of one carboxyl or amino group were less effective in initializing phase separation in POPC-membranes compared to derivatives that had modifications of both polar groups as well as the parent antibiotic. The effects were attributed to the restriction of the aggregation process by electrical repulsion between charged derivatives in contrast to neutral compounds. The significant correlation between the ability of derivatives to increase the permeability of model membranes—causing the appearance of single channels in lipid bilayers or inducing calcein leakage from unilamellar vesicles—and the minimal inhibitory concentration indicated that the antifungal effect of the conjugates was due to pore formation in the membranes of target cells.

## Introduction

In recent decades, the problem of treating infectious diseases caused by fungal infection has grown due to an increase in immunosuppressed patients and development of antibiotic resistance. Polyene macrolide antibiotics, such as amphotericin B and nystatin, have been widely applied in medicine to treat surface and deep mycoses due to their high activity [1]. In addition, resistance to polyenes develops very slowly.

It is believed that the mechanisms of action of amphotericin B (AmB) and nystatin are related to pore formation in the fungal cell membrane [2–10]. Unfortunately, due to their poor selectivity towards membranes of fungal *vs*. mammalian cells, polyene macrolides are the most toxic clinically used drugs, which substantially limits their pharmacological application [11,12]. Many attempts have been made to produce polyene derivatives and conjugates with reduced side effects [13–20]. Recently, benzoxaboroles, which are privileged structures in medicinal chemistry due to their desirable physicochemical and drug-like properties [21], were used for the synthesis of AmB-benzoxaborole hybrid antibiotics [22]. Some of these conjugates demonstrated promising antifungal activity and reduced toxicity against normal cells.

The aim of this work was to prove that semisynthetic derivatives based on AmB, including its benzoxaborole hybrids, have the same mode of action as the parent antibiotic, i.e., pore formation, and to compare the characteristics of ion channels formed by various derivatives.

## Materials and methods

All chemicals were of reagent grade. Synthetic 1,2-diphytanoyl-*sn*-glycero-3-phosphocholine (DPhPC), 1-palmitoyl-2-oleoyl-*sn*-glycero-3-phosphocholine (POPC), ergosterol (Erg) and 1,2-dipalmitoyl-*sn*-glycero-3-phosphoethanolamine-N-(lissamine rhodamine B sulfonyl) (Rh-DPPE) were obtained from Avanti Polar Lipids, Inc. (Pelham, AL). Amphotericin B (AmB, ***1***), calcein, Sephadex G-50, Triton X-100, EDTA, KCl, HEPES, NaOH, KOH and sorbitol were purchased from Sigma Chemical (St. Louis, MO). Water was distilled twice and deionized. Solutions of 2.0 M KCl were buffered using 5 mM HEPES-KOH at pH 7.0.

**Fig 1:**
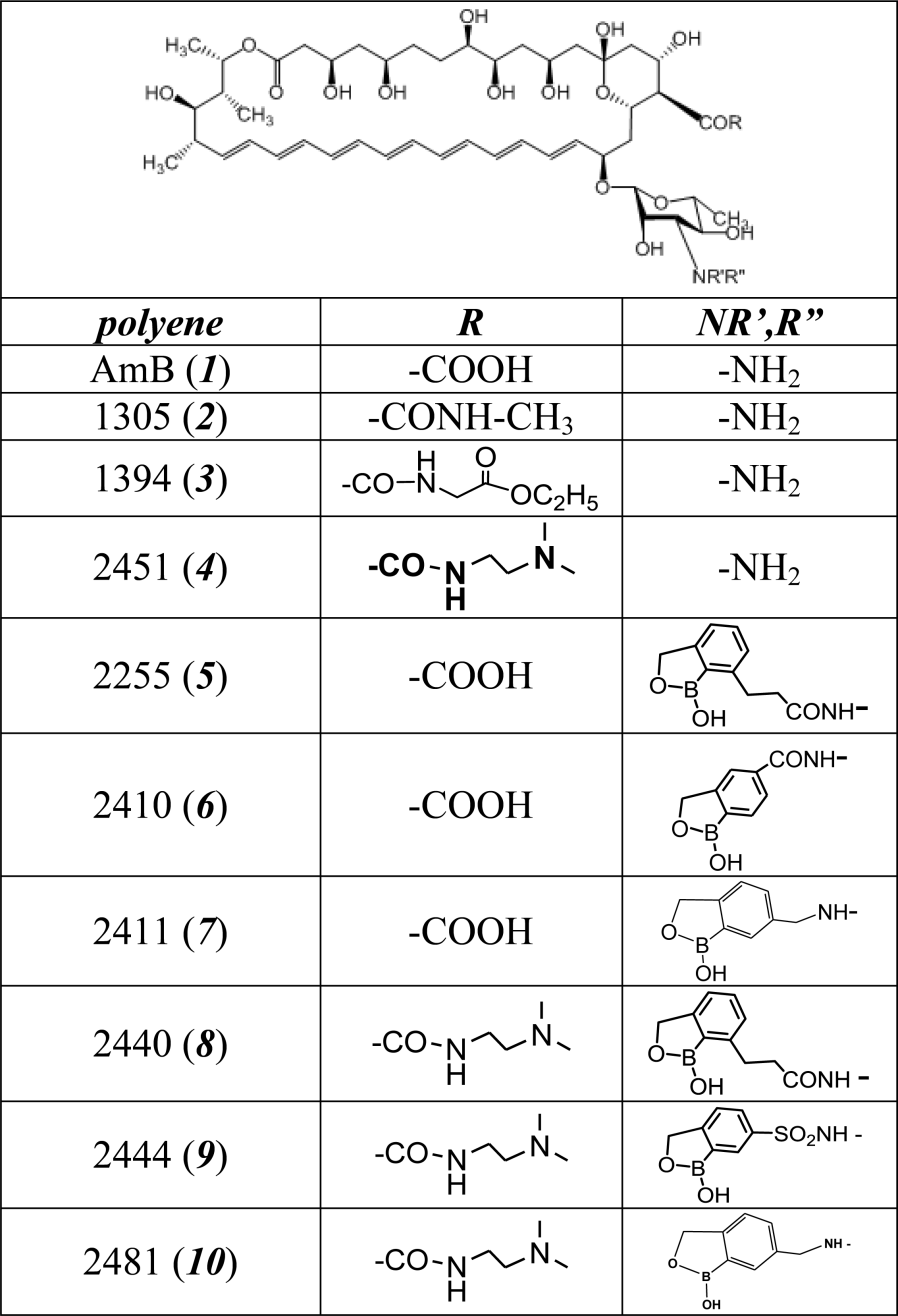
Chemical structure of AmB (*1*) and its conjugates *2* ÷ *10*.

The synthesis of polyene conjugates ***2*–*10*** is described in [22,23]. The chemical structures of the derivative compounds are shown in Fig 1.

### 1. Registration of ion channels in planar lipid bilayers

Virtually solvent-free planar lipid bilayers were prepared according to a monolayer-opposition technique [24] on a 50-*μ*m-diameter aperture in a10-*μ*m thick Teflon film separating two (*cis* and *trans*) compartments of the Teflon chamber. The aperture was pretreated with hexadecane. The lipid bilayers were made from 67 mol % DPhPC and 33 mol % Erg. After the membrane was completely formed and stabilized, various polyene conjugates (***2*–*10***) from a stock solution (1 mM in DMSO) were added to both compartments to obtain the final concentrations presented in the Table 1. Ag/AgCl electrodes with agarose/2 M KCl bridges were used to apply a transmembrane voltage (*V*) and measure the transmembrane current. “Positive voltage" refers to the case in which the *cis*-side compartment was positive with respect to the *trans*-side. All experiments were performed at room temperature. The final concentration of DMSO in the chamber did not exceed 10^−4^ mg/ml and did not produce any changes in the stability and conductance of the lipid bilayers.

**Table 1:** Characteristic parameters of the membrane activity of AmB (*1*) and its conjugates (*2*–*10*).

**polyene**	**C**_**Erg**_**, 10**^**−7**^ **M**	**C**_**Chol**_**/C**_**Erg**_	**MIC,** *μ***g/ml**^a^	P_So_, %	IF_max_, %	kinetic parameters		G_±200_, pS	[d(ln(I, pA)/d(│V│, mV)]∙10^−3^	τ, ms	P_op_
						t_1_, min	t_2_, min				
AmB (***1***)	1.1 ± 0.5	2.3 ± 1.2	0.5	90 ± 7	33 ± 9	0.18 ± 0.04	2.3 ± 0.4	11.2 ± 0.6^c^	11.6 ± 1.7	32 ± 2^c^	0.65 ± 0.08
1305 (***2***)	2.0 ± 0.6	–^b^	–^b^	43 ± 14	29 ± 5	–^d^	–^d^	17.7 ± 2.3	12.7 ± 2.5	12 ± 2	0.39 ± 0.09
1394 (***3***)	2.3 ± 1.0	–^b^	–^b^	44 ± 7	28 ± 4	0.18 ± 0.04	8.2 ± 3.5	22.4 ± 2.3	12.4 ± 2.6	15 ± 3	0.11 ± 0.06
2451 (***4***)	1.1 ± 0.3	2.4 ± 0.4	0.5	65 ± 10	31 ± 9	0.14 ± 0.01	8.5 ± 1.1	17.5 ± 2.9	13.0 ± 1.1	16 ± 5	0.20 ± 0.13
2255 (***5***)	4.9 ± 0.9	–^b^	4	54 ± 5	25 ± 5	0.93 ± 0.12	10.2 ± 0.8	16.4 ± 2.1	12.5 ± 2.2	12 ± 3	0.33 ± 0.14
2410 (***6***)	3.8 ± 1.7	–^b^	4	58 ± 7	24 ± 9	0.48 ± 0.12	11.3 ± 3.5	23.3 ± 2.4	13.8 ± 3.0	13 ± 3	0.23 ± 0.07
2411 (***7***)	1.6 ± 0.2	0.5 ± 0.2	1	67 ± 9	36 ± 6	0.21 ± 0.12	5.6 ± 2.3	26.0 ± 4.0	14.4 ± 2.8	15 ± 4	0.36 ± 0.18
2440 (***8***)	2.9 ± 0.8	5.4 ± 1.0	1	93 ± 10	33 ± 4	0.19 ± 0.07	8.6 ± 1.8	12.9 ± 2.9	12.0 ± 3.2	20 ± 3	0.28 ± 0.18
2444 (***9***)	2.7 ± 1.0	–^b^	1	86 ± 12	37 ± 3	0.17 ± 0.02	4.1 ± 0.6	13.2 ± 2.2	11.8 ± 2.4	20 ± 4	0.25 ± 0.11
2481 (***10***)	1.4 ± 0.3	–^b^	1	82 ± 12	34 ± 4	0.23 ± 0.07	4.6 ± 0.5	13.9 ± 2.9	11.9 ± 2.7	22 ± 5	0.32 ± 0.11

*C*_*Erg*_−the antibiotic threshold concentration required to observe single polyene channels in planar lipid bilayers composed of DPhPC:Erg (67:33 mol %), *C*_*Chol*_/*C*_*Erg*_*−*the ratio between antibiotic threshold concentrations required to observe single polyene channels in membranes composed of DPhPC:Chol and DPhPC:Erg (67:33 mol %); *MIC*–the lowest concentrations of agents that prevent visible growth of *Candida albicans; P*_*So*_−the percentage of POPC-liposomes with gel domains, *IF*_*max*_−the maximal leakage of calcein from unilamellar vesicles made from POPC:Erg (67:33 mol %), *kinetic parameters*–the characteristic times (fast component, *t*_*1*_, and slow component, *t*_*2*_) of two-exponential dependence fitting the time dependence of calcein release, *G*_*±200*_ –the mean conductance of polyene channels at ± 200 mV, d(ln*I*)*/*d*V*–a derivative of the function of dependence of the logarithm of the current flowing through the channel on transmembrane voltage, *τ*–the mean dwell time of polyene pores; *P*_*op*_*−*the probability of polyene channels to be in an open state.

a–according to [22]

b–the values were not determined

c–according to [5]

d–the two-exponential function does not fit the time dependence of calcein leakage induced by compound ***2***.

Current measurements were performed using an Axopatch 200B amplifier (Molecular Devices, LLC, Orlean, CA, USA) in the voltage clamp mode. Data were digitized by a Digidata 1440A and analyzed using a pClamp 10 (Molecular Devices, LLC, Orlean, CA, USA) and Origin 7.0 (OriginLab Corporation, Northampton, MA, USA). Current tracks were filtered by an 8-pole Bessel 100 kHz. Single-channel conductance (*G*) was defined as the ratio between the current flowing through a single polyene channel (*I*) and transmembrane potential (*V*). The total numbers of events used for the channel conductance fluctuation and dwell time analysis were 250 ÷ 500 and 1000 ÷ 3000, respectively. The probability of the polyene channel to be in an open state (*P*_*op*_) was determined as $\frac{\tau }{\tau +\tau_{close}}$, where *τ* is the dwell time of the single polyene channel and *τ*_*close*_ is the time that the channel is in a closed state.

### 2. Computational methods

Calculations of geometric parameters and partial charges were performed using the quantum-chemical semi empirical method AM1 [25] with the help of the Spartan-10 software package [26]. The calculations were performed with complete optimization of the geometric parameters; the initial approximation of the calculations was made using the molecular mechanics method.

### 3. The confocal fluorescence microscopy of giant unilamellar vesicles

Giant unilamellar vesicles (GUVs) were formed by the electroformation method on a pair of indium tin oxide (ITO) slides using a commercial Nanion vesicle prep pro (Munich, Germany) as previously described [27,28]. Lipid stock solutions of POPC were prepared in chloroform. Labeling was achieved by addition of the fluorescent lipid probe Rh-DPPE; its concentration in each sample was 1 mol %. The resulting aqueous liposome suspension containing 0.8 mM lipid and 0.5 M sorbitol was divided into 50 ml aliquots. Different polyene conjugates, ***2*–*10***, from the 10 mM DMSO stock solution were added to aliquots to a final concentration of 300 μM. The liposome suspension with polyene conjugates was allowed to equilibrate for 30 min at room temperature (25 ± 1°C). The sample was observed as a standard microscopy preparation, and 10 μl of the resulting liposome suspension without and with polyene conjugates was placed on a standard microscope slide and covered by a cover slip. GUVs were imaged through an oil immersion objective (100×/1.4HCX PL) using a Leica TCS SP5 confocal laser system Apo (Leica Microsystems, Mannheim, Germany). The temperature during observation was controlled by air heating/cooling in a thermally insulated camera.

Rh-DPPE clearly favors a liquid disordered phase (*l*_*d*_) and is excluded from the gel (*s*_*o*_) phase [29]. The percentage of vesicles (*p*_*i*_) with the respective phase separation type in each tested system was calculated as the ratio of phase-separated or homogenous GUVs to the total number of GUVs:

$$p_{i}=\frac{N_{i}}{N_{t}}\cdot 100\;\%,$$ (1)

where the *i*-type of the phase separation scenario in GUV indicates a homogeneously colored GUV in the *l*_*d*_-phase or liposomes with irregular uncolored *s*_*o*_−domains; *N*_*i*_−number of vesicles with the *i*-th type of the phase separation scenario (from 0 to 50); and *N*_*t*_−total number of counted vesicles in the sample (typically 50). At least 5–7 independent experiments were performed with each tested derivative.

### 4. Calcein release from large unilamellar vesicles

The fluorescence of calcein that leaked from large unilamellar vesicles (LUVs) was used to monitor the membrane permeabilization induced by AmB and its conjugates ***2****–****10***. LUVs were prepared from POPC:Erg (67:33 mol %) by extrusion using an Avanti Polar Lipid mini-extruder (Pelham, AL). The lipid stock in chloroform was dried under a gentle stream of nitrogen. A dry lipid film was hydrated by a buffer (35 mM calcein, 10 mM HEPES-NaOH, pH 7.4). The suspension was subjected to five freeze-thaw cycles and passed through a 100-nm Nuclepore polycarbonate membrane 13 times. Calcein that was not entrapped in vesicles was removed by gel filtration in a Sephadex G-50 column to replace the buffer outside the liposomes with a calcein-free solution (150 mM NaCl, 1 mM EDTA, 10 mM HEPES-NaOH, pH 7.4). The LUV suspension was diluted to obtain a total lipid concentration of 25 μM, which was assessed by a Phospholipids Assay Kit from Sigma Chemical (St. Louis, MO). The calcein in the vesicles fluoresced very poorly due to strong self-quenching at millimolar concentrations, while the fluorescence of the disengaged calcein in the surrounding media correlated with membrane permeabilization in the absence and presence of AmB and its conjugates ***2****–****10***.

AmB and its conjugates ***2*–*10*** from the stock solution (1 mM in DMSO) were added to calcein-loaded liposomes. Time-dependent calcein fluorescence de-quenching induced by 50 μM of AmB or its conjugates was measured over 35 min.

The degree of calcein release was determined at 25°C using a Fluorat-02-Panorama spectrofluorimeter (Lumex, Saint-Petersburg, Russia). A 10-mm quartz cuvette was used to measure calcein release from liposomes after the addition of AmB and its conjugates. The excitation wavelength was 490 nm, and the emission wavelength was 520 nm. Addition of Triton X-100 from a 10 mM water solution to a final concentration of 0.1 M to each sample led to complete disruption of LUVs, and the intensity of fluorescence after releasing the total amount of calcein from liposomes was measured.

The relative intensity of calcein fluorescence (*IF*, %) was used to describe the dependence of the permeabilization of the liposomes on the type of membrane-active compound. *IF* was calculated using the following formula:

$$IF=\frac{I-I_{0}}{I_{\max}/0.9-I_{0}}\cdot 100\%,$$ (2)

where *I* and *I*_*0*_ are the calcein fluorescence intensities in the sample in the presence and in the absence of polyene, respectively, and *I*_*max*_ is the maximal fluorescence of the sample after lysis of liposomes by Triton X-100. A factor of 0.9 was introduced to calculate the dilution of the sample by Triton X-100.

## Results and discussion

**Fig 2:**
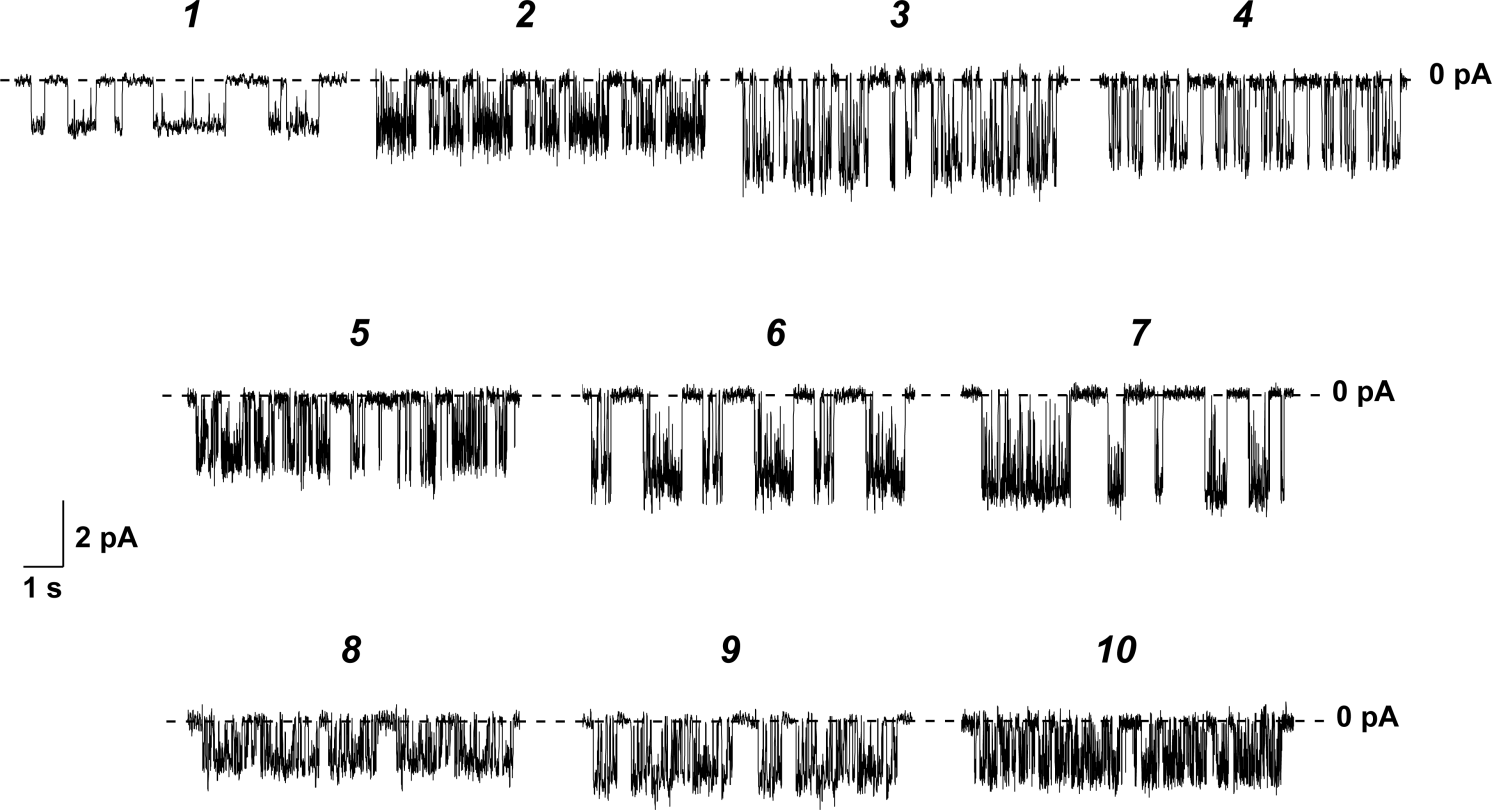
Current fluctuations corresponding to openings and closures of single channels induced by AmB (*1*) and compounds ## 1305 (*2*), 1394 (*3*), 2451(*4*), 2255 (*5*), 2410 (*6*), 2411 (*7*), 2440 (*8*), 2444 (*9*), and 2481 (*10*). The lipid bilayers compared to DPhPC:Erg (67:33 mol %) and bathed in 2.0 M KCl (pH 7.4). The transmembrane voltage was –150 mV.

**Fig 3:**
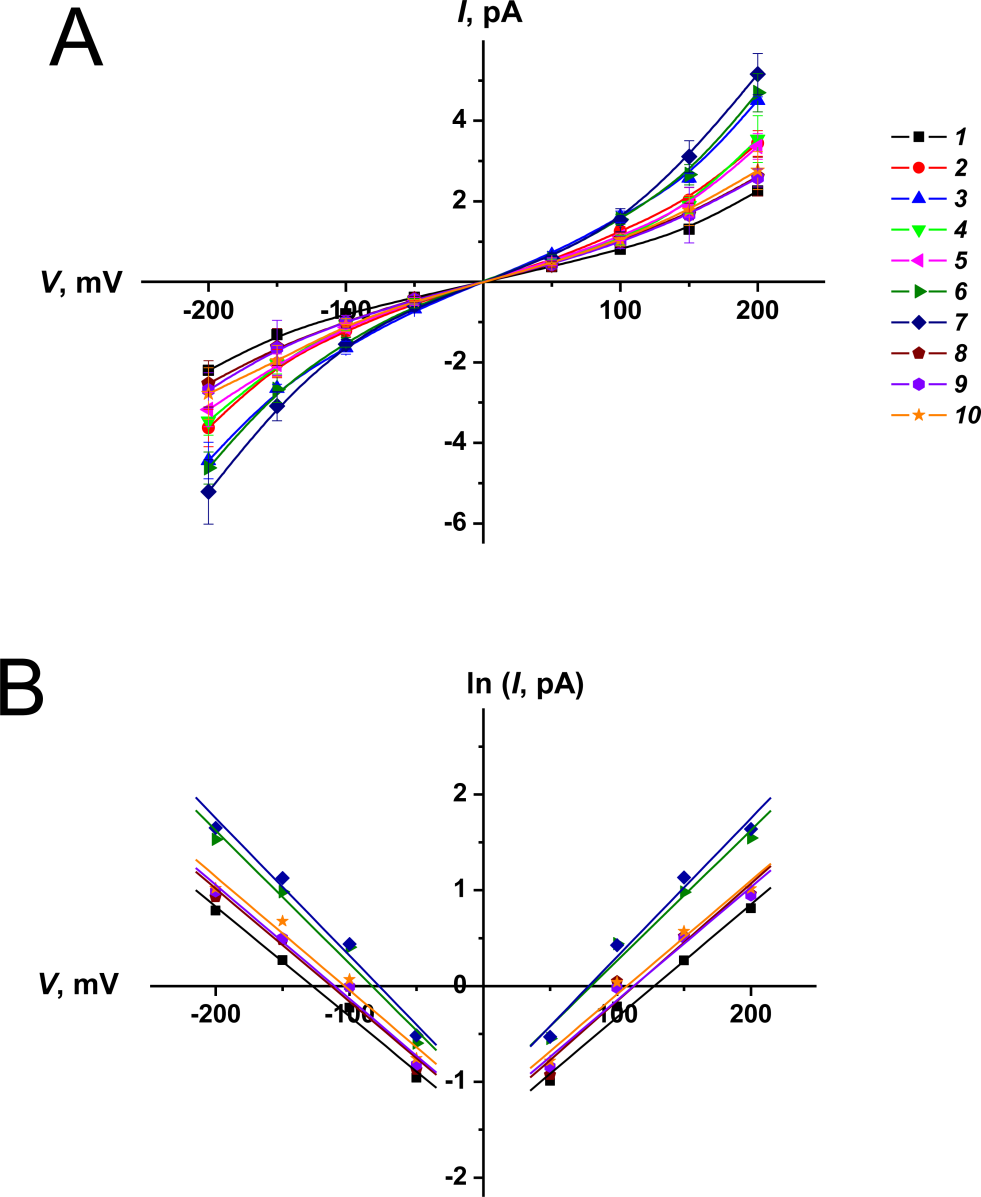
(**A**)–***I*–*V* curves of single channels produced by AmB (*1*) and its conjugates (*2* ÷ *10*).** Membranes were made from DPhPC:Erg (67:33 mol %) and bathed in 2.0 M KCl (pH 7.4). (**B**)–**Dependence of logarithm of current flowing through channels formed by AmB (*1*) and its derivatives (*6*, *7*, *8*, *9*, *10*) on transmembrane voltage**.

Fig 2 presents current fluctuations corresponding to openings and closures of single channels formed by AmB (***1***) and its conjugates ***2****–****10*** in lipid bilayers composed of DPhPC and ergosterol (67:33 mol %) in 2 M KCl (pH 7.4) at –150 mV. The tested derivatives produce pores of larger amplitude than AmB does. Fig 3A shows *I–V* diagrams of pores produced by different antibiotics. AmB channels induced by the addition of polyene to both sides are characterized by symmetric superlinear *I*(*V*)-curves. Chemical modification of natural polyenes does not affect the symmetry of the *I*(*V*)-diagram. However, the pores induced by some derivatives demonstrate the enhanced dependence of current flowing through the channel on the transmembrane voltage, which is easier to visualize in *I–V* diagrams in the linearized form, ln*I*(*V*) (Fig 3B). Table 1 summarizes the mean conductance at ± 200 mV and slope coefficients of lines approximating the ln*I*(*V*)-dependences of the pores formed by AmB (***1***) and its conjugates ***2****–****10***. The channel conductance increases in the order ***1*** ≤ ***8*** ≈ ***9*** ≈ ***10*** ≤ ***5*** ≤ ***2*** ≈ ***4*** ≤ ***3*** ≈ ***6*** ≤ ***7***. In the latter case, the conductance exceeds the conductance of the natural channel by a factor of more than two. The coefficient d(ln*I*)*/*d*V*, which characterizes the voltage dependence of pore conductance, increases in the order ***1*** ≤ ***8*** ≈ ***9*** ≈ ***10*** ≤ ***3*** ≈ ***5*** ≤ ***2*** ≈ ***4*** ≤ ***6*** ≤ ***7***. The ability to enhance the voltage dependence of the current is in good agreement with the efficacy of increasing the pore conductance. The data obtained contradict the general belief that modification of the charged groups has almost no influence on the conductance of the channel, while it strongly depends on the structure of the polar chain and is related to the orientation of the hydroxyl groups of the lactone ring [30–32]. According to Borisova et al. [31] the symmetric double-length channel induced by the addition of AmB to both sides has an intrinsic anion affinity that is determined by the positively charged hydrogens of the ligand OH-dipoles. According to Khutorsky [32], in a half-pore there are two ligand sites that make a major contribution to anion coordination: (OH)_3_ and (OH)_4_ hydrogens at the wide entrance and (OH)_6_ and (OH)_7_ ligands at the narrow part. Using the quantum-chemical semi empirical method AM1, we evaluated the partial charges of the hydrogens of these ligand groups of AmB and its derivatives (Table 2). One can see that charges on the indicated hydrogens increase in the order ***1*** ≤ ***8*** ≈ ***9*** ≈ ***10*** < ***2*** ≈ ***3*** ≈ ***4*** ≈ ***5*** < ***6*** ≤ ***7***. The row is in a good agreement with the conductance changes. Thus, the first experimental evidence was obtained of the significant effect of the chemical modification of the charged groups in the head of the AmB molecule on the translocation barrier to the anion through the channel formed by polyene. The correlations between the hydrogen charge and d(ln*I*)*/*d*V*-coefficients might indicate that the voltage dependence of pore conductance is determined by the OH-dipoles of the lactone ring of the polyene molecule.

**Table 2:** The partial charges of hydrogens in ligand (OH)-groups of AmB (*1*) and its conjugates *2* ÷ *10*.

ligand group	AmB (*1*)	1305 (*2*)	1394 (*3*)	2451 (*4*)	2255 (*5*)	2410 (*6*)	2411 (*7*)	2440 (*8*)	2444 (*9*)	2481 (*10*)
(OH)_3_	0.210	0.268	0.268	0.267	0.269	0.452	0.453	0.226	0.225	0.226
(OH)_4_	0.212	0.262	0.262	0.262	0.262	0.427	0.427	0.222	0.222	0.222
(OH)_6_	0.210	0.247	0.247	0.246	0.246	0.399	0.407	0.208	0.208	0.208
(OH)_7_	0.225	0.266	0.266	0.266	0.266	0.422	0.431	0.227	0.227	0.227

The results obtained with channels induced by one-side addition of AmB and its derivatives are presented in the Supporting Information. The half-pore of AmB is a cation-selective rectifier [4,33,34]. S1 Fig shows *G–V* diagrams of single-length pores produced by AmB and its conjugates ***2****–****10*** in lipid bilayers composed of DPhPC and ergosterol (67:33 mol %) in 2 M KCl (pH 7.4). The conductance of polyene single-length channels at 200 mV increases in the order ***8*** < ***1*** ≈ ***5*** ≈ ***9*** ≈ ***3*** ≈ ***10*** ≈ ***6*** ≈ ***2*** ≤ ***4*** ≤ ***7***. In a half-pore, there is one ligand site that makes a major contribution to cation coordination: (OH)_6_ and (OH)_7_ oxygens [32]. S1 Table summarizes the values of the partial charges of the oxygens: the absolute values increase in the order ***8*** ≈ ***9*** ≈ ***10*** ≤ ***1*** < ***2*** ≈ ***3*** ≈ ***4*** ≈ ***5*** < ***6*** ≤ ***7***. Despite the incomplete correspondence of the row and changes in the conductance of single-length channels—which might be because computer simulation is performed in a vacuum in the absence of an electric field—a common trend is observed. Moreover, the row is in agreement with the results obtained for double-length channels. Therefore, the same mechanisms are responsible for the conductance changes on both symmetric and asymmetric channels induced by AmB and its derivatives.

In Fig 2, channels induced by the addition of AmB derivatives to both sides more rapidly flicker between the open and closed states than pores produced by the parent antibiotic. It is known that the stabilization of the channel complex in an open state is caused by electrostatic interactions between the ammonium and carboxyl groups of adjacent antibiotic molecules [35]. Moreover, AmB’s NH^3+^-group is involved in a specific interaction with the PO^4−^-group of zwitterionic lipid molecules and the sterol hydroxyl group and thus adds their contributions to the stability of AmB complexes [36]. Thus, the energy of hydrogen bond interactions between neighboring antibiotic molecules is diminished due to the loss of one or both polar group charges during chemical modification, and this, along with a similar in-mechanism reduction of the interactions between the NH^3+^-group from AmB and PO^4−^-group of zwitterionic lipid molecules and ergosterol hydroxyl group promotes destabilization of the AmB open pore. Our results are in agreement with the data obtained in [30], which suggest that the neutralization of one or both charges of the AmB molecule (both by chemical modification and by pH shift) decreases the probability of the channel to be in the conducting state. Table 1 presents the mean dwell times, τ, and probability of the ion channels formed by AmB and its hybrids to be in an open state, *P*_*op*_. The lifetime increases in the order ***2*** ÷ ***7*** ≤ ***8*** ÷ ***10*** < ***1***. Thus, neutralization of one of the polar group charges provides a more pronounced effect than modification of both groups. It is likely that electrostatic repulsion between AmB molecules that have the same charge destabilizes the channel, while neutral antibiotic molecules are more prone to aggregate. Uncharged but bulky substituents prevent interaction between AmB molecules and destabilize the conducting complex.

**Fig 4:**
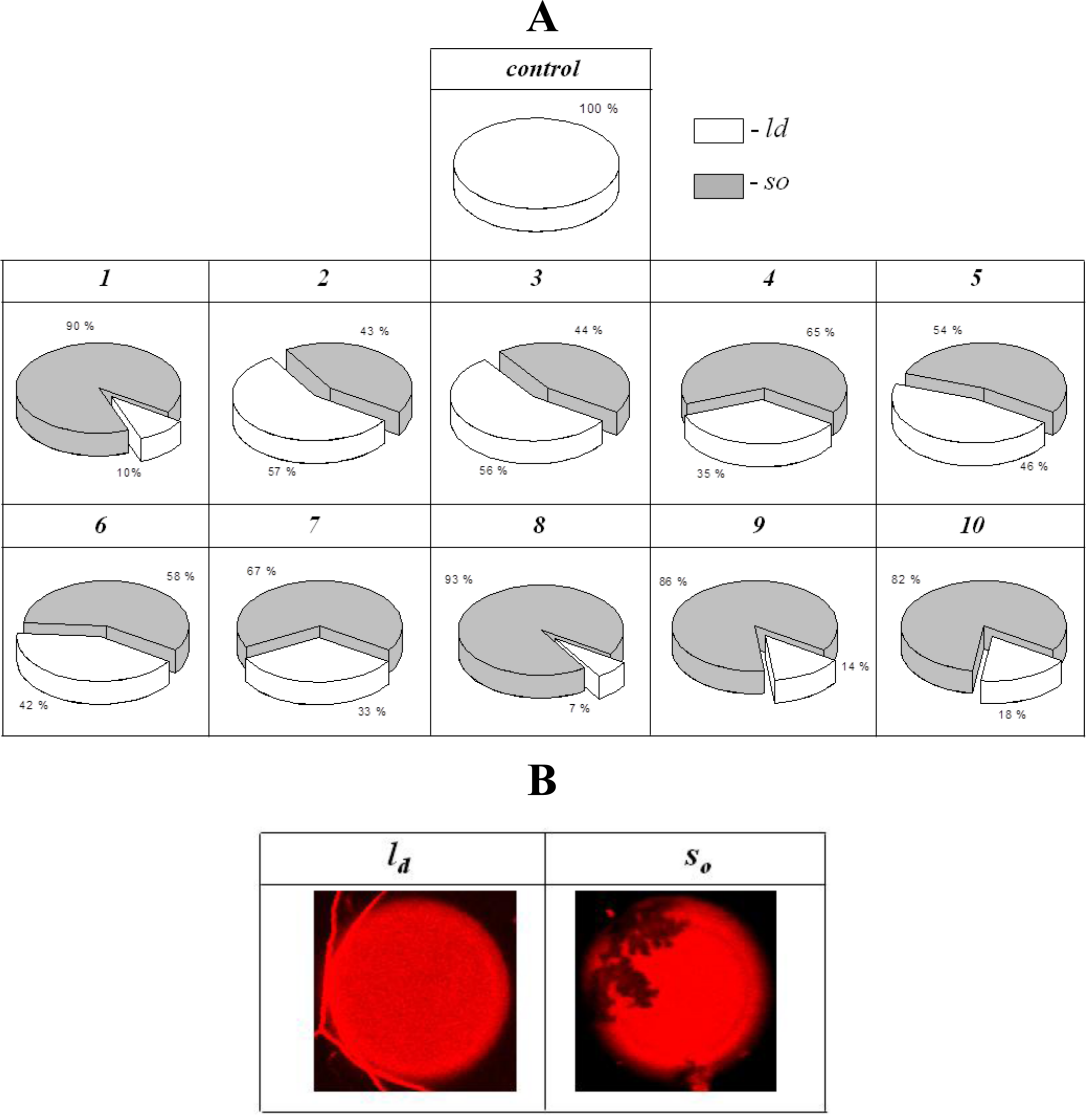
(A)–Percentages of giant unilamellar POPC-vesicles characterized by different types of phase separation (sector related to relative number of homogeneously colored vesicles in liquid-disordered phase (*l*_*d*_) is white; sector presented percentage of liposomes with gel domains (*s*_*o*_) is gray) in the absence (control) and presence of 300 μM of polyenes in the liposome solution: AmB (*1*), and compounds ## 1305 (*2*), 1394 (*3*), 2451(*4*), 2255 (*5*), 2410 (*6*), 2411 (*7*), 2440 (*8*), 2444 (*9*), and 2481 (*10*). (B)–Fluorescence micrographs of POPC-liposomes demonstrating different types of phase separation (*l*_*d*_, *s*_*о*_) in the presence of 300 μM compounds #1305 (*2*). Size of each image is 26 μm× 26 μm.

Fig 4A shows pie charts that demonstrate the percentage of phase-separated vesicles composed of POPC after introduction of various polyene conjugates into a liposome suspension, *P*_*So*_. Notably, pure POPC does not produce a gel phase at room temperature (its melting temperature is near –2°C [37]). Addition of polyene to a liposome suspension produces a phase separation in the membranes [8,26], supporting the assumption that polyenes can directly interact with phospholipids [38–43]. The appearance of gel domains that exclude the fluorescent marker of the fluid disordered phase is observed (Fig 4B). From Fig 4A, one can notice the different ability of polyene hybrids to aggregate and immobilize the phospholipids. Compounds ***2*** ÷ ***7*** (with modification in one carboxyl or amino group) are less effective in initializing phase separation in POPC-membranes compared to derivatives that are simultaneously modified by both polar groups (***8*** ÷ ***10***) and the parent antibiotic (***1***). One simple hypothesis to explain these findings is that the appearance of the total integral charge in the head of the antibiotic, as a result of the chemical modification of one amino or carboxyl group, prevents the self-assembly of polyene molecules and their segregation into a separate phase; by contrast, aggregation of zwitterionic AmB molecules and neutral analogs in the membrane is not restricted by electrical repulsion. The good correlation between *P*_*So*_ and τ-values (Table 1) demonstrates their relation to the aggregation state of the antibiotics.

**Fig 5:**
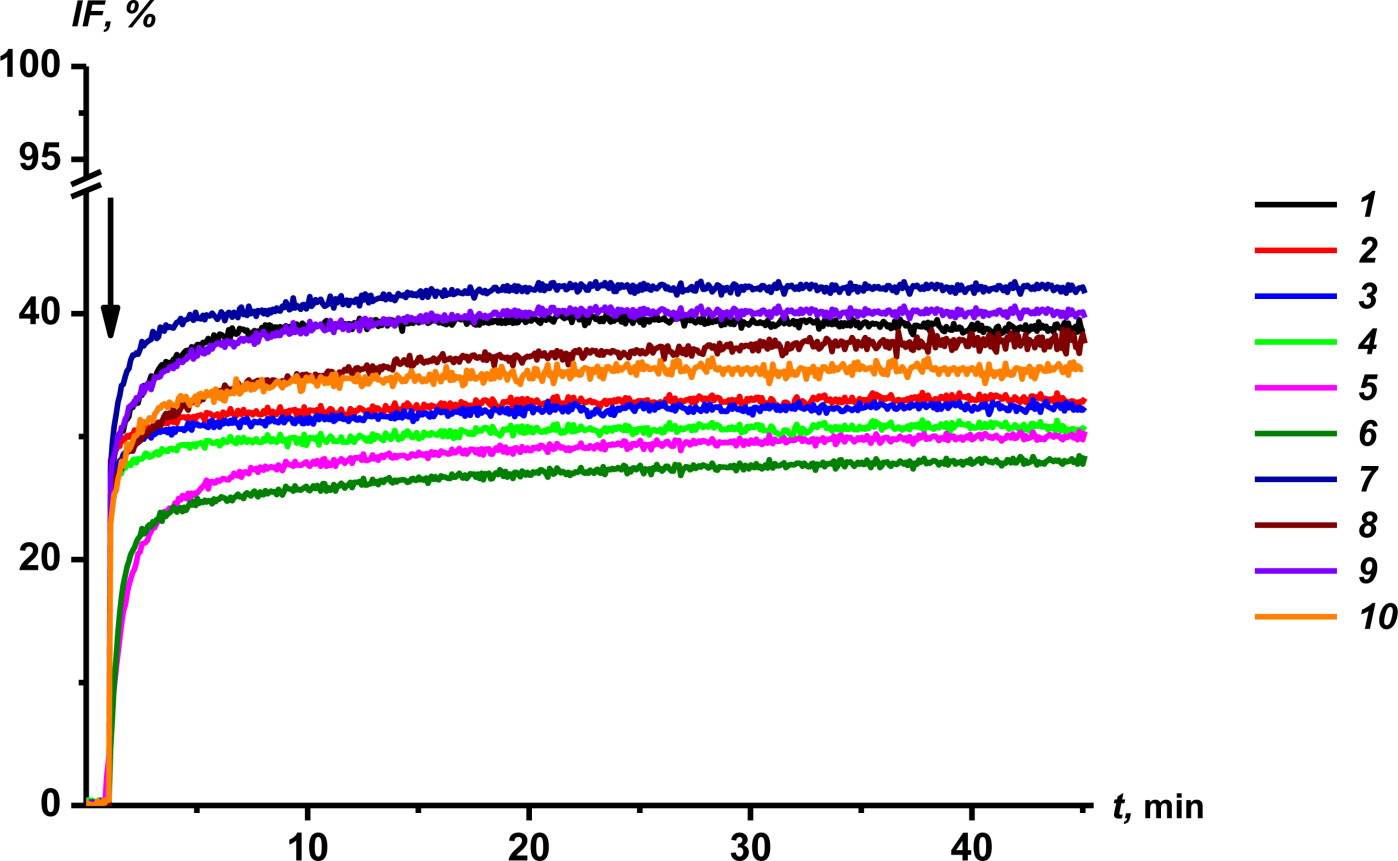
Time dependence of relative fluorescence of calcein (*IF*, %) leaked from POPC:Erg (67:33 mol %) vesicles. The moment of addition of AmB (***1***) or its conjugates (***2*** ÷ ***10***) into a liposomal suspension up to 50 μM is indicated by an arrow.

Fig 5 demonstrates the ability of AmB and its hybrids to lead to calcein efflux from large unilamellar vesicles prepared from POPC admixed with ergosterol (67:33 mol %), mimicking the conditions found in natural biomembranes (i.e., introduction of a polyene antibiotic from outside of the cell membrane). Two-exponential dependences are used to fit the time dependences of the calcein release induced by AmB and its conjugates. The characteristic parameters of the dependences; maximal leakage, *IF*_*max*_; and time related to fast and slow components, *t*_*1*_ and *t*_*2*_, respectively, are presented in Table 1. One can conclude that derivatives ***5*** and ***6*** have the lowest efficiency to disengage fluorescent markers from liposomes: minimal *IF*_*max*_-values and maximal values *t*_*1*_ and *t*_*2*_. The data obtained are in agreement with the measured threshold concentration of antibiotics required to observe single polyene channels in DPhPC:Erg-bilayers, *C*_*Erg*_ (Table 1). Moreover, the threshold concentrations of compounds ***5*** and ***6*** required to inhibit the growth of *Candida albicans* are four-fold larger than those AmB and derivatives ***4*** and ***7*** ÷ ***10*** [22]. The significant correlation between the MICs and ability of derivatives to increase membrane permeability (*C*_*Erg*_ and characteristic parameters of kinetics of calcein leakage) indicates that the antifungal effect of the conjugates is due to pore formation in the membranes of target cells.

In this case, the *in vivo* efficacy is determined by the total transmembrane current induced by polyene (*I*_*P*_) and is proportional to the current flowing through the single polyene pore, which is a nonlinear function of transmembrane voltage (*I*(*V*)) (Fig 3A), and the number of open pores (*N*_*op*_), which does not depend on the transmembrane voltage in the case of polyene channels (data not shown). *N*_*op*_ is determined by the number of channel precursors (*N*_*pr*_) and the probability of the channel to be in an open state (*P*_*op*_) (Table 1). As the 6÷8 polyene molecules form the functional channel [2,44–46], *N*_*pr*_ should be proportional to the antibiotic concentration in the 6÷8th degree. Thus, the effective concentration of the antibiotic is the main contributor to *I*_*P*_. For this reason, a significant correlation between *C*_*Erg*_ and *MIC*-values is observed, but it is not noticeable between the other parameters that characterize the pore-forming activity of the tested antibiotics (*G*, d(ln*I*)*/*d*V*, τ, and *P*_*op*_) and *MIC*-values.

AmB is toxic to mammalian cells and is known to be a hemolytic, which greatly complicates its use for pharmacological purposes. According to [22], the hemolytic activities of the AmB derivatives and AmB were different. The percentage of hemolysis relative to water (positive control, 100%) in AmB derivatives **5, 6** and **8** was lower (6–7%) than that of AmB (23%). Thus, new macrolide polyene antibiotics (AmB derivatives) have an advantage over AmB in the *in vitro* experiments. Additionally, to support these findings, we evaluated the ratio of the threshold concentrations of polyenes in Chol- and Erg-containing bilayers (Table 1). For a higher ratio, the toxicity of the antibiotic should be lower, and the *C*_*Chol*_/*C*_*Erg*_-value of the AmB derivative **8** is higher than AmB. In addition, antibiotic derivative AmB **8** can be recommended as a leading compound for further investigation as a prospective drug due to it water solubility. Future investigations into the antifungal activity and toxicity of the leading compounds in animals will reveal agents with the highest efficacy and lowest toxicity.

## Conclusions

In lipid bilayers, the tested AmB amides and its conjugates with benzoxaboroles produced pores with larger amplitudes and shorter lifetimes than AmB. The conductances of the pores are related to the changes in the partial charges of OH-group hydrogens, which determined the anion coordination in the channel. Neutralization of one of the polar group charges in the polyene head had a pronounced effect on diminishing the channel lifetime and initializing membrane phase separation than modifying both groups. The effects are related to the restriction of the aggregation of charged compounds. The correlation between the derivative ability to increase the permeability of lipid bilayers and MIC indicated that the antifungal effect was due to pore formation in target cell membranes. It should also be mentioned that some of the tested derivatives are characterized by a reduced ability to form pores in cholesterol- vs. ergosterol-containing membranes compared to the parent antibiotic. This finding makes new AmB derivatives quite promising for pharmacological development.

## Supporting information

S1 Fig *G–V* curves of the single channels produced by one-side addition of AmB (*1*) and its conjugates (*2* ÷ *10*). (PDF)

S1 Table The partial charges of oxygens in ligand (OH)-groups of AmB (*1*) and its conjugates *2* ÷ *10*. (PDF)

## Abbreviations

AmB: amphotericin B
DPhPC: 1,2-diphytanoyl-*sn*-glycero-3-phosphocholine
POPC: 1-palmitoyl-2-oleoyl-*sn*-glycero-3-phosphocholine
Erg: ergosterol
