# Supplementary material for: Pore-forming activity of new conjugate antibiotics based on amphotericin B
Source: PLoS One. 2017 Nov 29;12(11):e0188573. doi: 10.1371/journal.pone.0188573 (PMC5706719; doi:10.1371/journal.pone.0188573)
Supplement: S1 Fig — (PDF) [file pone.0188573.s001.pdf]

Supporting information

Single-length channels formed by amphotericin B and its derivatives

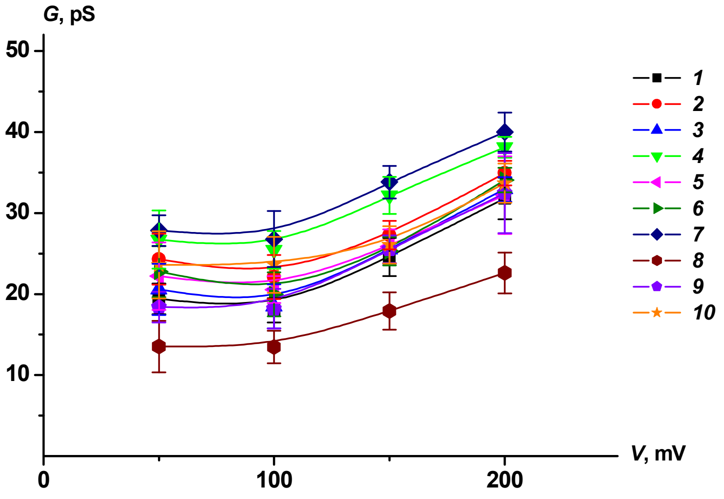

S1 Fig. *G–V* curves of the single channels produced by one-side addition of AmB (*1*) and its conjugates (*2* ÷ *10*). The membranes were made from DPhPC:Erg (67:33 mol %) and bathed in 2.0 M KCl (pH 7.4).
