# Supplementary material for: Pore-forming activity of new conjugate antibiotics based on amphotericin B
Source: PLoS One. 2017 Nov 29;12(11):e0188573. doi: 10.1371/journal.pone.0188573 (PMC5706719; doi:10.1371/journal.pone.0188573)
Supplement: S1 Table — (PDF) [file pone.0188573.s002.pdf]

Supporting information

S1 Table. The partial charges of oxygens in ligand (OH)-groups of AmB (*1*) and its conjugates *2 ÷ 10*.

ligand group	AmB (*1*)	1305 (*2*)	1394 (*3*)	2451 (*4*)	2255 (*5*)	2410 (*6*)	2411 (*7*)	2440 (*8*)	2444 (*9*)	2481 (*10*)
$(OH)_6$	-0.345	-0.391	-0.391	-0.391	-0.390	-0.689	-0.709	-0.342	-0.341	-0.341
$(OH)_7$	-0.341	-0.389	-0.389	-0.390	-0.390	-0.712	-0.718	-0.338	-0.339	-0.338
